# MicroRNA Expression Levels Are Altered in the Cerebrospinal Fluid of Patients with Young-Onset Alzheimer’s Disease

**DOI:** 10.1007/s12035-018-1032-x

**Published:** 2018-03-30

**Authors:** Paul M. McKeever, Raphael Schneider, Foad Taghdiri, Anna Weichert, Namita Multani, Robert A. Brown, Adam L. Boxer, Anna Karydas, Bruce Miller, Janice Robertson, Maria Carmela Tartaglia

**Affiliations:** 1 Tanz Centre for Research in Neurodegenerative Diseases, Krembil Discovery Tower, 60 Leonard Ave., 4KD-481, Toronto, ON M5T 2S8 Canada; 2 Laboratory Medicine and Pathobiology, University of Toronto, Toronto, ON Canada; 3 Institute for Medical Sciences, University of Toronto, Toronto, ON Canada; 4 University Health Network, Toronto, ON Canada; 5 Montreal Neurological Institute, McGill University, Montreal, QC Canada; 6 Memory and Aging Center, University of California, San Francisco, San Francisco, CA USA

**Keywords:** Alzheimer’s disease, Young-onset, Cerebrospinal fluid, Exosomes, MicroRNA

## Abstract

Clinical diagnosis of Alzheimer’s disease (AD) prior to the age of 65 years is classified as young-onset (YOAD), whereas diagnosis after the age of 65 years is considered late-onset (LOAD). Although rare autosomal mutations more commonly associate with YOAD, most YOAD and LOAD cases are sporadic. YOAD and LOAD share amyloid and tau pathology, but many YOAD patients show increased disease severity and rate of progression. The current study examined the microRNA (miRNA) expression profile from exosomes isolated from the cerebrospinal fluid (CSF) of YOAD patients with biomarker-confirmed AD. Results uncovered miR-16-5p, miR-125b-5p, miR-451a, and miR-605-5p as differentially expressed in the CSF-derived exosomes of YOAD patients when compared with healthy controls (HC). In a cohort of LOAD patients, miR-125b-5p, miR-451a, and miR-605-5p were similarly altered in expression, but miR-16-5p showed similar expression to control. Analysis of the mRNA targets of these miRNAs revealed transcripts enriched in biological processes relevant to the post-mortem posterior cingulate cortex transcriptome in YOAD from a previously published microarray study, including those related to neuron projections, synaptic signaling, metabolism, apoptosis, and the immune system. Hence, these miRNAs represent novel targets for uncovering disease mechanisms and for biomarker development in both YOAD and LOAD.

**Electronic supplementary material:**

The online version of this article (10.1007/s12035-018-1032-x) contains supplementary material, which is available to authorized users.

## Introduction

Young-onset Alzheimer’s disease (YOAD) occurs before the age of 65 years and represents up to 10% of all AD cases. Fewer than 1% of AD are caused by autosomal dominant mutations [1–3]; hence, the majority of AD cases are sporadic regardless of age at disease onset [4, 5]. Although an age of 65 years for delineating YOAD versus late-onset AD (LOAD) was selected based on social factors such as the traditional retirement age [6], key imaging, neuropathological, and neurochemical differences based on an age cutoff of 65 years have been reported in the literature. YOAD patients present with more severe gray matter atrophy [7], more abundant senile plaques, neurofibrillary tangles, and synaptic loss [8], as well as greater deficits in acetylcholine [9] than LOAD patients. YOAD patients appear more likely to present with impaired language, attention, and visuospatial function, compared with LOAD patients who experience more memory deficits [10].

In terms of rate of progression, some reports have shown a faster rate of cognitive decline in younger patients [8, 10–12]; others found no association between the age of onset and rate of decline [13–15]. Currently, biomarker protein levels of amyloid β-42 (Aβ_42_), total tau (t-tau), and phosphorylated tau (p-tau) in the cerebrospinal fluid (CSF) of YOAD and LOAD patients are routinely used to confirm a diagnosis consistent with AD [16–19]. Some studies have reported a higher level of CSF t-tau and p-tau in YOAD compared with LOAD, suggesting a more severe disease and more rapid decline [20, 21]. However, this difference in CSF tau levels is inconsistently found [22]. Other novel biomarkers that relate to disease mechanism are of great interest in YOAD as current biomarkers of amyloid and tau do not provide differentiating features between YOAD and LOAD.

MiRNAs are stable, small, non-coding RNAs (21–23 nucleotides) involved in the degradation and/or translational repression of target messenger RNAs (mRNAs) (reviewed in [23]). Up to 2588 mature human miRNAs (miRNAs) have been identified (miRbase.org). Individual miRNAs can target and silence up to thousands of mRNAs, and multiple miRNAs can target single genes (reviewed in [24]). Cell-to-cell communication is considered a key role for miRNAs since they are released into the extracellular milieu by several mechanisms, including being complexed with Argonaute [25, 26] and lipoprotein particles [27] and packed into small extracellular vesicles called exosomes for exchange of genetic material between cells [26, 28]. Exosomes in particular represent a stable and enriched source of miRNAs in biofluids such as the blood [29, 30] and CSF [31]. Importantly, exosomes have been implicated in cell-to-cell communication within the central nervous system. Exosomes secreted from neurons can regulate the brain vasculature [32], and exosomes secreted from astrocytes can modulate synaptic plasticity [33, 34]. In AD, neuronal exosomes may be involved in Aβ_42_ release as a result of early endosomal maturation [35] but may also be involved in intracerebral uptake of Aβ_42_ [36]. Exosomes derived from AD and Down syndrome blood contain lower Aβ_42_ and increased p-tau [37] reflecting the changes seen in CSF of AD [16]. Hence, we reasoned that specifically exploring miRNA expression profiles in exosomes would inform on AD-relevant disease mechanisms.

Generally, studies elucidating the miRNA expression profile in the CSF of AD patients are currently surging in the literature [38]. In the majority of these studies, LOAD was compared with healthy controls [39–43]; others compared LOAD with healthy controls and other neurological diseases [44–46], while LOAD was also examined against other forms of dementia [47, 48]. Whole CSF was examined in most cases, whereas two of these studies profiled for miRNA changes in CSF-derived exosomes from LOAD patients. The first study used TaqMan miRNA arrays (746 human miRNAs) to profile for changes in the CSF from Parkinson’s disease (PD) and LOAD patients relative to controls [46]. While several candidate miRNAs were uncovered for both PD and LOAD, validation by independent real-time PCR was only conducted on the PD cohort. In the second study [41], LOAD was compared with healthy controls in whole CSF using Exiqon’s human miRNome panels (752 human miRNAs) followed by examination of candidates in CSF-derived exosomes. Several candidate miRNAs were altered in LOAD compared with controls. In CSF-derived exosomes, the detectability of miRNAs increased, especially when testing the same candidates that were already identified in the whole CSF screen. However, the specific, high-throughput miRNA profile changes occurring in the CSF-derived exosomes of YOAD remain to be elucidated.

The current study explores the miRNA expression profile in exosomes derived from the CSF of biomarker-confirmed sporadic YOAD patients compared with that in healthy controls (HC). Here, we uncovered a decrease in miR-16-5p, miR-451a, and miR-605-5p and an increase in miR-125b-5p in YOAD patients versus HC. Combining the relative expression of these four miRNAs by regression analysis effectively distinguished YOAD relative to HC. Interestingly, the four miRNAs altered in YOAD share common targets and pathways altered in the post-mortem YOAD brain [49]. In a cohort of LOAD patients, we showed that miR-451a and miR-605-5p were similarly decreased and miR-125b-5p increased in LOAD but there was no significant difference in miR-16-5p expression compared with HC. Hence, these results revealed three miRNAs with altered expression in the CSF-derived exosomes of both YOAD and LOAD. The YOAD-specific decrease of exosomal miR-16-5p provides a potential candidate involved in disease mechanisms related to YOAD.

## Materials and Methods

### Patient and Clinical Assessment

All patients with AD were seen at the University Health Network (UHN) Memory Clinic (Toronto Western Hospital) between 2011 and 2016 and diagnosed with possible or probable AD (McKhann, 2011). The cognitively normal healthy control CSF samples (*n* = 12) were obtained from UHN (*n* = 2) and University of California San Francisco Memory and Aging Center (*n* = 10). The YOAD group comprised patients aged less than 65 years (*n* = 17) and the LOAD group greater than 65 years (*n* = 13). The lumbar punctures were performed according to ADNI protocol [50], and CSF was collected in polypropylene tubes. A clinical AD diagnosis was confirmed using the CSF protein biomarkers: CSF Aβ_42_, p-tau, and t-tau levels. Innogenetic assays were run, and the results were considered consistent with AD diagnosis if p-tau > 68 pg/ml and Aβ_42_ to t-tau index (ATI) < 0.8 [18, 51, 52]. Patients also underwent cognitive assessment using either the Montreal Cognitive Assessment [53] or modified Behavioural Neurological Assessment [54].

### Isolation of Exosomal miRNA from CSF

Exosomal preparations were performed using the miRCURY™ Exosome Isolation Kit (Exiqon) following the manufacturer’s instructions. To prepare each sample, 1.1 ml of undiluted input CSF was centrifuged for 5 min at 3000×*g* to pellet cell debris and 1.0 ml of the CSF supernatant was used as input for exosome extraction. High-quality miRNA was isolated from each exosome prep along with appropriate spike-in controls (Exiqon). Next, column purification was performed using the miRCURY RNA Isolation Kit following the manufacturer’s instructions (Exiqon).

### Discovery Phase: High-Throughput PCR with Exiqon Human miRNome Panels I + II

Complementary DNA (cDNA) was synthesized using the locked nucleic acid (LNA) Universal cDNA Synthesis Kit (Exiqon). The ExiLENT SYBR Green 2X Master Mix (Exiqon) was used to prepare cDNA samples for amplification and visualization by quantitative real-time PCR (qrt-PCR). For each sample, cDNA was added to the SYBR master mix and was loaded at 10 μl per well across Exiqon human miRNome panels I + II (V4.M, Exiqon), which are 2 × 384-well plates consisting of a total of 752 well-established miRNA human primer sets. Both 384-well plates were run in tandem on a 7900HT thermocycler (Applied Biosystems, Life Technologies).

### Quality Control, Normalization, and Statistical Analyses

For data filtering and quality control or individual reactions, raw amplification and melting curve data obtained for both Exiqon human panels I + II on the 7900HT thermocycler were imported into the Thermo Fisher Cloud Relative Quantification (RQ) app (Thermo Fisher Scientific, https://apps.thermofisher.com/apps/dashboard/#). Through automated processing and visual inspection across plates, only reaction wells displaying linear amplification, Ct values < 39, and that passed a melt curve analysis were included in subsequent analysis. Subsequently, all human panel data from both YOAD (*n* = 17 × 2 plates) and HC (*n* = 12 × 2 plates) was simultaneously imported into the GenEx software (6.0) for sample-to-sample (inter-plate) calibration. The selection of miRNAs for normalization was performed using established algorithms geNorm [55] and Normfinder [56]. From this, the spike-ins UniSp6 and cel-miR-39-3p as well as the stably and highly expressed endogenous miR-204-5p were used to normalize across all Ct values using a combined geometric mean of all three Ct values [55]. A previous study showed miR-204-5p to be the most abundant miRNA, with highly stable expression in whole CSF (see Tables 4 and 5 in [57]). An ANOVA followed by pairwise comparisons was performed after normalization. Additional candidate miRNAs for validation were uncovered using presence/absence of signal data mining followed by Fisher’s exact test for significance. Visualization of relative miRNA expression was performed using custom R scripts and heatmap.2 function in the R package gplots (v3.0.1) and GraphPad Prism v7.0c.

### Validation Phase: Individual Primer Set qrt-PCR

In the same YOAD (*n* = 17) and HC (*n* = 12) samples as the discovery, individual qrt-PCR reactions were performed to validate differentially expressed miRNAs in YOAD that were identified in the discovery phase. Validated miRNAs in YOAD were examined in a cohort of LOAD patients (*n* = 13). For each miRNA primer set, three technical replicates per sample were included. Using the same cDNA and SYBR prep as discussed above, qrt-PCR reactions were performed using individual LNA primer sets (Exiqon) on 96-well plates using the ABI Step One Plus Real-Time PCR System (Applied Biosystems, Life Technologies). A list of all primer sets used in the current study is in Supplementary Table 1. Data normalization was performed similarly as above with the geometric mean of UniSp6 and miR-204-5p. Relative miRNA expression changes were calculated as relative expression to control using the Ct value from each qrt-PCR reaction by the 2^−ΔΔCt^ method [58, 59]. Statistical significance between groups was performed using one-way ANOVA followed by Bonferroni correction. All bar graphs were drawn in GraphPad Prism v7.0c.

### Discrimination Analysis

Receiver operating characteristics (ROC) analysis was performed to evaluate the capacity of each individual validated miRNA to distinguish either YOAD or LOAD from HC. ROC analysis is an established statistical approach for assessing the diagnostic potential of a continuous clinical variable [60, 61]. In a ROC analysis, for a biomarker of disease, the cutoff for correctly identifying patients is called sensitivity (true positive rate) and is plotted against the specificity (false positive rate) to produce a ROC curve. To assess combinatorial performance of validated miRNAs, a generalized linear model (GLM) was fitted with the relative expression data from combinations of validated miRNAs. The clinical diagnosis for YOAD or LOAD was given binary outcomes (0 or 1, respectively), where the predicted probability was modeled with a binomial distribution and logit function [62]. GLM data was produced using basic R (v3.3.2) functions. ROC curve probabilities, area under the curve (AUC), and CI were calculated using the R package pROC [63]. Finally, k-fold cross validation of each GLM was performed using adapted code from the R package DAAG [64]. All AUC data was estimated using the trapezoidal method, and all 95% confidence intervals (CI) for the probabilities calculated at each cutoff were calculated in R (v3.4.0) using bootstrap sampling [65] with 1000 bootstrap replicates.

### MicroRNA Target Prediction

For miRNAs confirmed in the validation phase, target prediction was performed using TargetScan v7.1 [66]. TargetScan predicts miRNA gene targets by considering both canonical and non-canonical miRNA binding sites on target mRNA using experimentally backed datasets. The overlap between miRNA targets was visualized using the R package venn (v1.2), which requires the R package QCA [67]. Next, the tissue location of enriched expression for targets of validated miRNAs was determined using FunRich v3.0 which combines multiple established databases including UniProt, Human Protein Atlas, Human Proteome Browser, Human Proteome Map, ProteomicsDB, and Human Proteinpedia to infer regional and cell-type enrichment of target mRNAs for a given list of miRNAs [68].

### Analysis of Published Microarray Data

Published raw microarray data from a post-mortem study that contrasted the posterior cingulate cortex (PCC) of sporadic YOAD (*n* = 7) and HC (*n* = 7) was retrieved from GEO accession ID GSE39420 [49]. Several R packages available through the Bioconductor framework [69] were used to analyze the microarray data. A detailed pipeline for conducting a differential expression analysis on Affymetrix microarray data in R [70] was modified and applied here. Briefly, the Affymetrix fluorescent intensity data was normalized using the R package affy (v1.52.0) [71] and a differential expression analysis was performed using the R package limma (v3.30.13) [72] to distinguish the PCC of sporadic YOAD versus HC. The false discovery rate (FDR) was set at *q* < 0.05. Pathway analysis of overlapping transcripts was performed using the Kyoto Encyclopedia of Genes and Genomes (KEGG) pathway data from Database for Annotation, Visualization, and Integrated Discovery (DAVID v6.8) [73].

### Gene Set Enrichment Analysis and Enrichment Map Visualization

Enrichment of Gene Ontology (GO) biological process (BP), cellular compartment (CC), and molecular function (MF) terms [74] was determined using Gene Set Enrichment Analysis (GSEA; v2.1.0) [75]. *Homo sapiens* GO BP, CC, and MF gene sets without inferred electronic annotation from the February 2018 release were retrieved from the online repository available at http://download.baderlab.org/EM_Genesets/ [76]. A log_2_ fold change ranked list of differentially expressed sporadic YOAD (*n* = 7) versus HC (*n* = 7) was imported into GSEA, and a GO BP analysis was performed. Results from the GO BP GSEA were imported into the Cytoscape [77] (v3.3.0) plug-in entitled Enrichment Map [78] (v2.1.0) to visualize GO BP, MF, and CC term themes in the data as a network of nodes and edges. AutoAnnotate was used to create clusters around redundant GO terms between nodes using similarity coefficients [79]. To select the most overrepresented terms to report on the enrichment map, WordCloud [80] was used. FDR was set to *q* < 0.001 for all analyses. Gene sets were pre-ranked using GSEA.

## Results

### Cerebrospinal Fluid Donor Characteristics

The CSF donor demographic data is summarized in Table 1. The mean age at LP of the HC cohort (*n* = 7 females, *n* = 5 males) was 66.5 ± 7.7, and all HC were confirmed biomarker-negative for AD (data not shown). The YOAD patients (*n* = 10 females; *n* = 7 males) had a mean age of onset of 56.8 ± 4.9 years. The duration of disease was 3.9 ± 2.3 years, whereby CSF was obtained by LP at an average age of 60.9 ± 4.6 years. The YOAD group showed protein biomarker levels consistent with AD (Aβ_42_ = 356.0 ± 159.1 pg/ml; total tau = 744.5 ± 375.0 pg/ml; phospho-tau = 101.7 ± 37.9 pg/ml; ATI = 0.37 ± 0.22). The ApoE4 genotype distribution in YOAD was as follows: 52.94% had zero alleles, 35.29% had one allele, and 11.76% had two alleles.

**Table 1:** Cerebrospinal fluid donor demographic data

Patient	Demographic	Data
Healthy controls	*N*	12
	Gender (female/male)	7/5
	Age at time of LP (years)^a^	66.5 ± 7.7
Young-onset AD	*N*	17
	Gender (female/male)	10/7
	Age of onset (years)^a^	56.8 ± 4.9
	Age at time of LP (years)^a^	60.9 ± 4.6
	Disease duration (years)^a^	3.9 ± 2.3
	MoCA^b^	
	*N* completed	14
	Age at time of testing (years)^a^	61.36 ± 4.7
	Score (/30)^a^	12.1 ± 6.7 (min 3, max 20)
	Revised BNA^c^	
	*N* completed	12
	Age at time of testing (years)^a^	59.83 ± 4.5
	Total score (/329)^a^	136.7 ± 62.7 (min 55, max 255)
	Orientation (/12)^a^	6.5 ± 2.3
	Memory immediate recall (/30)^a^	8.9 ± 4.6
	Delayed recall (/27)^a^	2.8 ± 4.4
	Delayed recognition (20)^a^	15.3 ± 3.7
	Visuospatial (/32)^a^	15.7 ± 10.7
	Executive function (/123)^a^	39.1 ± 31.6
	Language (/85)^a^	48.3 ± 17.8
	Aβ_42_ (pg/ml)^a^	356.0 ± 159.1
	Total tau (pg/ml)^a^	744.5 ± 375.0
	Phospho-tau (pg/ml)^a^	101.7 ± 37.9
	ApoE (*N*)	3 3 (8); 3 4 (6); 4 4 (2); 2 3 (1)
Late-onset AD	*N*	13
	Gender (female/male)	5/8
	Age at time of LP (years)^a^	75.5 ± 4.6
	Disease duration (years)^a^	3.6 ± 2.7
	Aβ_42_ (pg/ml)^a^	431.3 ± 139.4
	Total tau (pg/ml)^a^	721.6 ± 245.1
	Phospho-tau (pg/ml)^a^	97.1 ± 19.7
	ApoE (*N*)	3 3 (6); 3 4 (6); 2 3 (1)

^a^Mean ± standard deviation

^b^Montreal Cognitive Assessment

^c^Behavioural Neurology Assessment

For the LOAD patients (*n* = 5 females; *n* = 8 males), the mean age at LP was 75.5 ± 4.6 years. The protein biomarker levels for the LOAD group were also consistent with AD (Aβ-42 = 431.3 ± 139.4 pg/ml; total tau = 721.6 ± 245.1 pg/ml; phospho-tau = 97.1 ± 19.7 pg/ml; ATI = 0.37 ± 0.17). In this case, prevalence of the ApoE4 genotypes in LOAD patients was 53.85% with zero alleles, 46.15% with one allele, and no patients with two alleles. The LOAD group presented with more disease comorbidities than the YOAD group (Supplementary Table 2). Overall, an average of 1.4 (min 0, max 5) disease comorbidities was observed across all YOAD patients with seven patients showing no comorbidities (Supplementary Table 2). LOAD patients showed an average of 2.6 (min 0, max 6) disease comorbidities, and only one patient showed no comorbidities (Supplementary Table 2). These results are consistent with previous findings showing that LOAD patients are likely to present with more disease comorbidities than YOAD [81, 82].

### Discovery Phase: the miRNA Expression Profile from CSF-Derived Exosomes Is Altered in YOAD

**Fig. 1:**
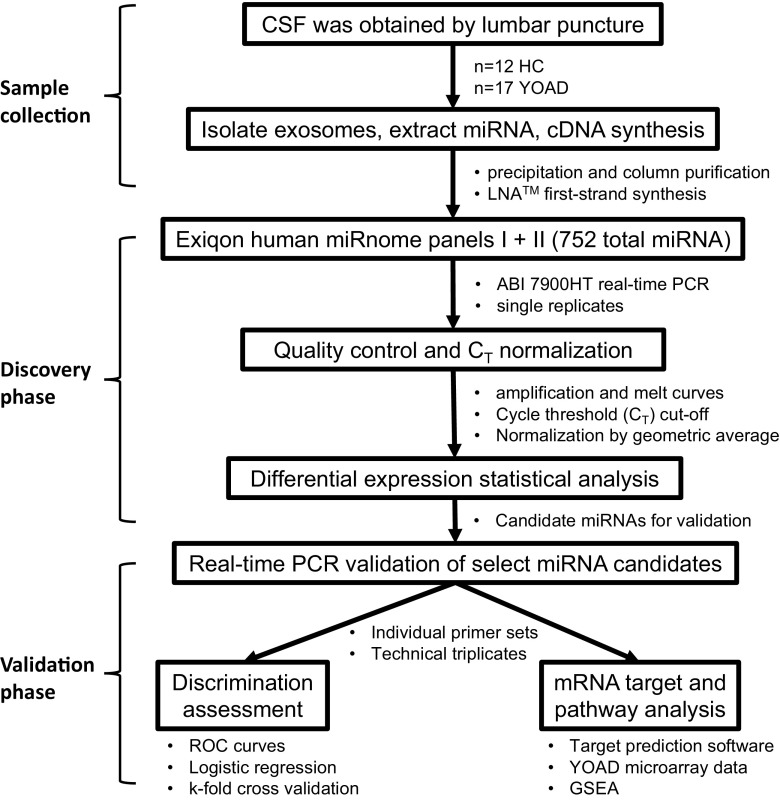
Workflow for the identification of altered miRNAs in the exosomal cerebrospinal fluid obtained from young-onset Alzheimer’s disease patients versus healthy controls. Pipeline of three phases: CSF sample collection phase, discovery phase, and validation phase. CSF cerebrospinal fluid, YOAD young-onset Alzheimer’s disease, HC healthy controls, LNA locked nucleic acid. Applied Biosystems (ABI) 7900 real-time PCR; receiver operating characteristics (ROC); Gene Set Enrichment Analysis (GSEA) of Gene Ontology Biological Processes terms

**Fig. 2:**
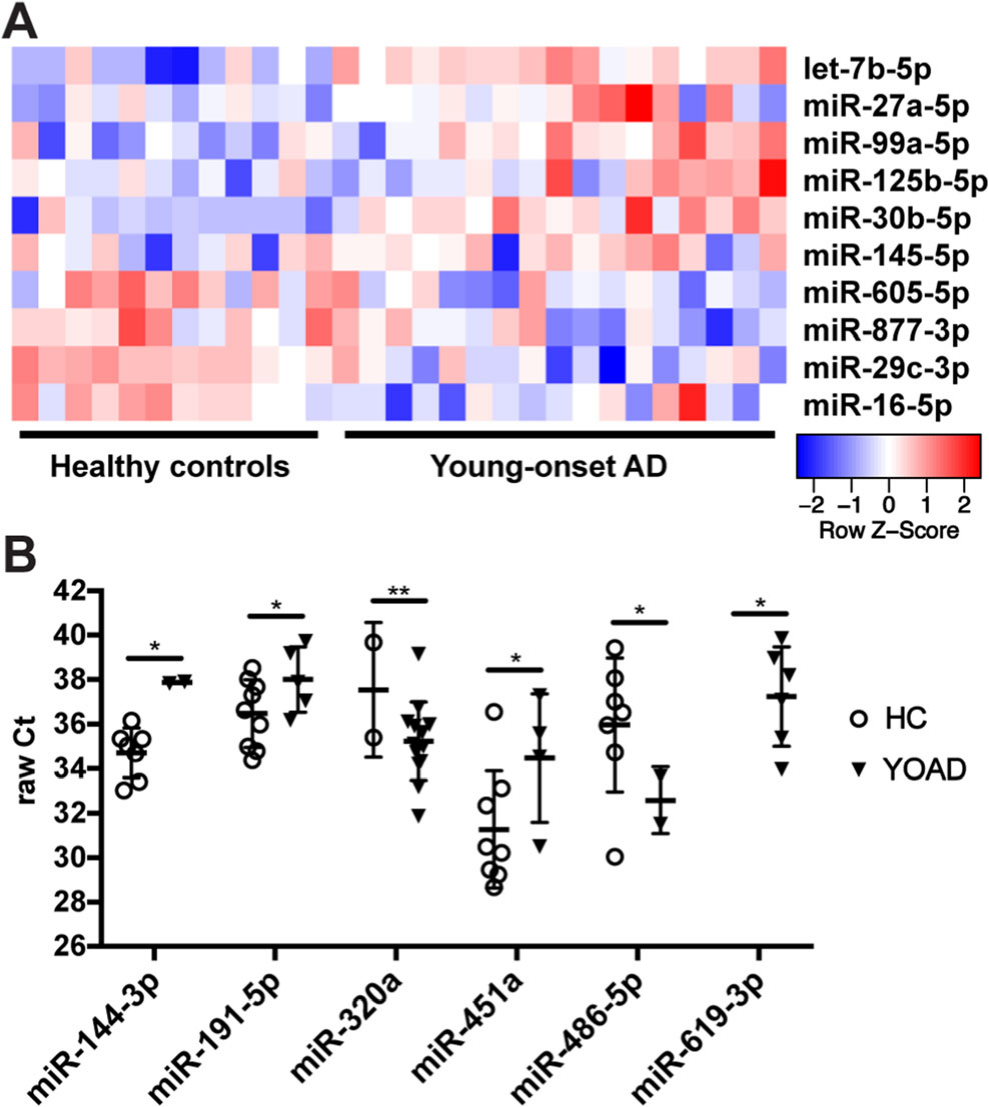
Discovery phase reveals candidate miRNAs altered in the CSF of YOAD patients versus healthy controls. **a** Heatmap of differentially expressed miRNAs in the CSF of YOAD patients (*n* = 17) versus HC (*n* = 12) identified using one-way ANOVA and pairwise comparisons (*p* < 0.05). Relative expression level normalized using the geometric mean of spike-ins UniSp6 and cel-miR-39-3p and the endogenous miR-204-5p. Rows are sorted by decreasing fold-change relative to HC. **b** Six additional candidate microRNAs uncovered in the discovery phase using presence/absence of expression mining and Fisher’s exact test for determining significance. *p < 0.05; **p < 0.01

The workflow for identifying differentially expressed miRNAs in the exosomes from CSF obtained from YOAD patients is in Fig. 1. Raw data from the high-throughput qrt-PCR human miRNome panels I + II underwent quality control measures as described in the “Material and Methods” section. After quality control and applying a Ct cutoff of < 39, a total of 164 miRNAs were detected across all 29 samples with a 14% detection floor (Supplementary Table 3). For normalization, the Ct values for spike-ins UniSp6 (Supplementary Fig. 1a) and cel-miR-39-3p (Supplementary Fig. 1b) along with the endogenous miR-204-5p (Supplementary Fig. 1c) were combined to generate the geometric mean (Supplementary Fig. 1d) using established protocols [55, 56]. In total, 48 miRNAs were detected at two thirds of all samples. From this list, an ANOVA followed by pairwise comparisons uncovered an increase in six and decrease in five candidate miRNAs in YOAD versus HC using uncorrected comparisons (Fig. 2a; all *p* < 0.05). Increased miRNAs included let-7b-5p, miR-27a-5p, miR-99a-5p, miR-125b-5p, miR-30b-5p, and miR-145-5p, whereas decreased miRNAs included miR-605-5p, miR-877-3p, miR-29c-3p, and miR-16-5p. Further candidate miRNAs were uncovered across remaining miRNAs using Fisher’s exact test whereby miR-144-3p, miR-191-5p, miR-451a, and miR-486-5p (all *p* < 0.05) were less commonly detected in YOAD cases, whereas miR-320a (*p* < 0.01) and miR-619-3p (*p* < 0.05) were more commonly detected in YOAD (Fig. 2b).

### Validation Phase: Distinct miRNAs Distinguish YOAD or LOAD from Healthy Controls

**Fig. 3:**
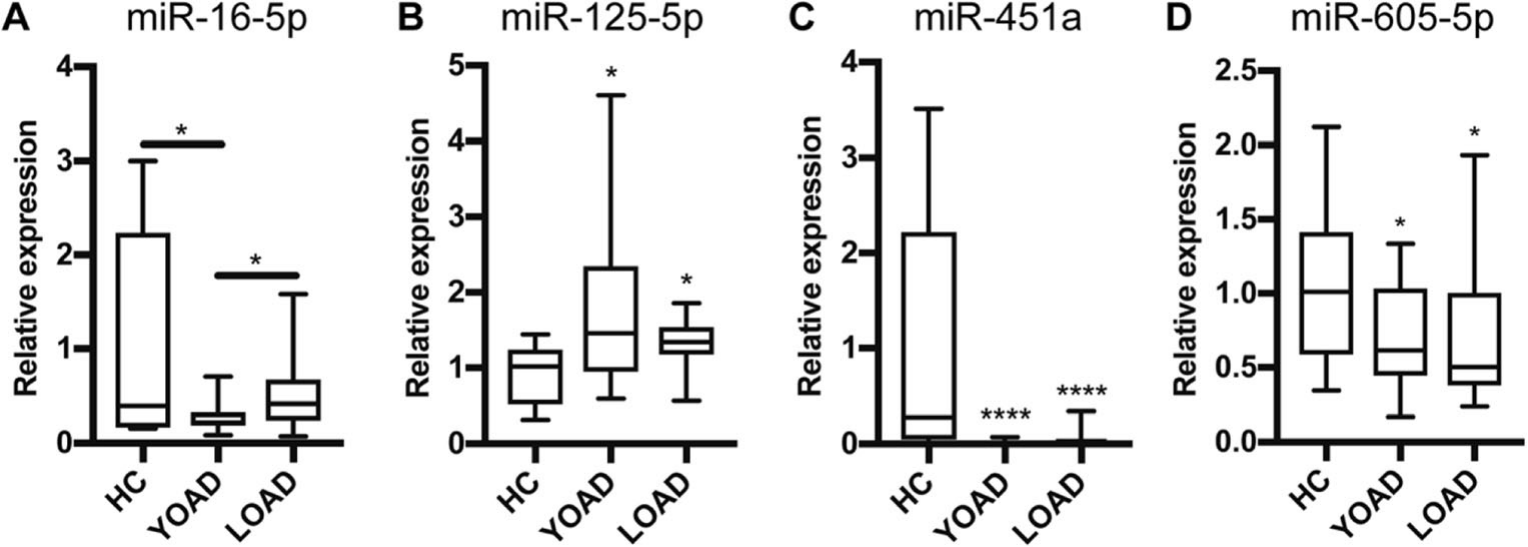
Validation phase uncovers miRNAs altered in the CSF of YOAD patients versus healthy controls. Independent validation of discovery phase results with quantitative real-time PCR showing relative expression in YOAD (*n* = 17) versus HC (*n* = 12) for **a** miR-16-5p, **b** miR-125b-5p, **c** miR-451a, and **d** miR-605-5p. Significance between groups was determined using ANOVA followed by pairwise comparisons with Bonferroni correction. *p < 0.05; ****p < 0.0001

We validated our results from the discovery phase in the same cohort of YOAD and HC using qrt-PCR in technical triplicates. We then examined whether validated miRNAs in YOAD were also altered in LOAD. For quality control of individual assays, the qrt-PCR efficiency and melt curves for each validated miRNA are provided (summarized in Supplementary Fig. 2). Similar to the discovery phase, the geometric mean of the cycle threshold for spike-in UniSp6 (Supplementary Fig. 3a) and endogenous miR-204-5p (Supplementary Fig. 3b) was used to normalize qrt-PCR data across groups (Supplementary Fig. 3c). An ANOVA followed by corrected pairwise comparisons showed a decrease of miR-16-5p in YOAD but not LOAD (Fig. 3a, *p* < 0.05), an increase of miR-125b-5p in both YOAD and LOAD (Fig. 3b, *p* < 0.05), a robust decrease of miR-451a in both YOAD and LOAD (Fig. 3c, *p* < 0.0001), and a decrease of miR-605-5p in YOAD and LOAD versus HC (Fig. 3d, *p* < 0.05). A pairwise comparison between YOAD and LOAD for miR-16-5p was significantly different (Fig. 3a, *p* < 0.05) suggesting a YOAD-specific decrease in CSF levels of exosomal miR-16-5p relative to LOAD. Additionally, the differential expression of miR-125b-5p, miR-451a, and miR-605-5p was found to be in common between YOAD and LOAD.

Next, we asked whether the expression of the validated miRNAs differed based on the age at which the LP was performed. Pearson correlation analysis was performed for the relative expression of all four validated miRNAs versus age at LP. We found that there was a significant negative correlation for miR-125b-5p expression in HC with age (Supplementary Fig. 4a; *R*^2^ = 0.624, *p* = 0.0022), but this effect was insignificant in YOAD (Supplementary Fig. 4b; *R*^2^ = 0.0055, *p* = 0.777) and LOAD (Supplementary Fig. 4c; *R*^2^ = 0.624, *p* = 0.0022). Hence, this suggests that expression of miR-125b-5p decreases with age in HC but may remain at high expression levels in AD regardless of age. Although there was a potential trend toward a positive correlation of miR-451a in LOAD (Supplementary Fig. 4c; *R*^2^ = 0.304, *p* = 0.063) and miR-605-5p in HC (Supplementary Fig. 4c; *R*^2^ = 0.2599, *p* = 0.091) and LOAD (Supplementary Fig. 4c; *R*^2^ = 0.263, *p* = 0.088), no other miRNAs showed an age effect.

**Fig. 4:**
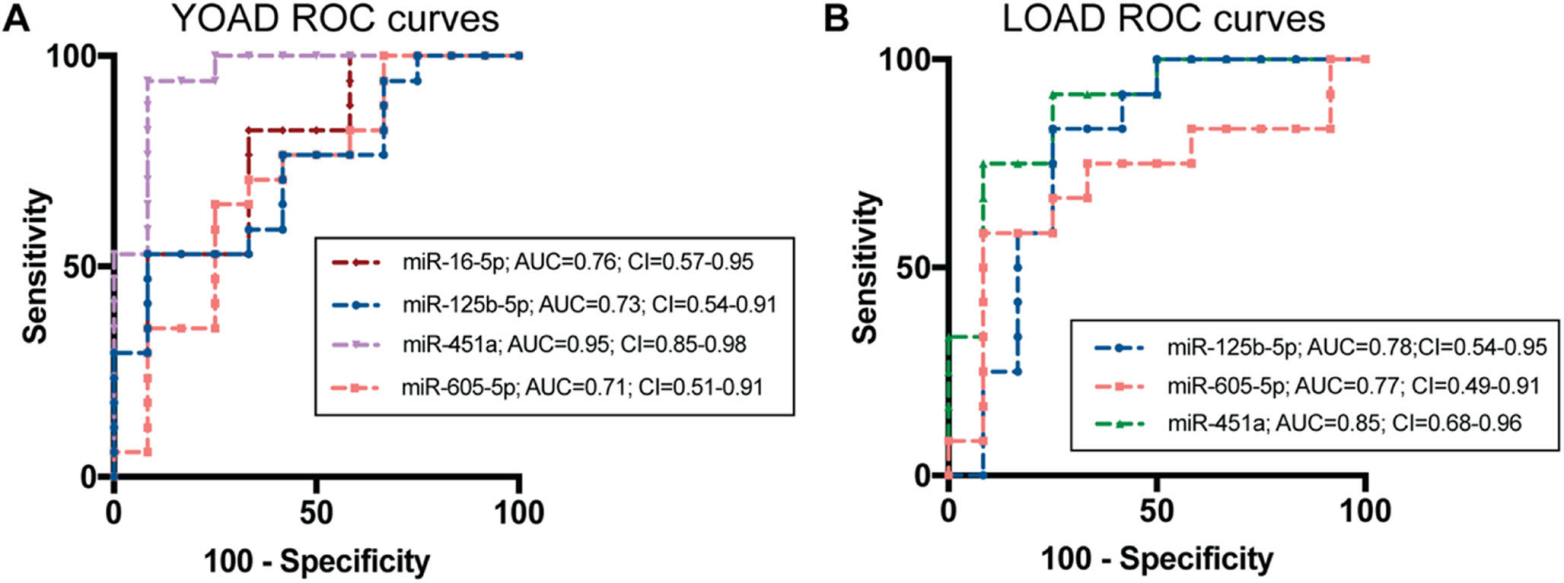
Performance of miRNAs at distinguishing YOAD or LOAD patients from healthy controls. Receiver operating characteristics (ROC) curves plotted as true positive rate (sensitivity) versus false positive rate (100 − specificity) for **a** YOAD and **b** LOAD. Area under the curve (AUC) was calculated using the trapezoid method, and the confidence intervals (CI) were calculated using bootstrap sampling in R (v3.4.0)

ROC analysis for HC versus YOAD was performed on the relative expression data for each of the validated miRNAs and is shown in Fig. 4a. From this, the ROC curve for miR-16-5p in YOAD showed an AUC = 0.760 and CI = 0.572−0.948, miR-125b-5p showed an AUC = 0.723 and CI = 0.537−0.914, and miR-605-5p showed an AUC = 0.706 and CI = 0.501−0.911. The miR-451a (AUC = 0.951, CI = 0.855−0.982) showed robust performance at distinguishing YOAD from HC. These results indicate that the clinical potential of these miRNAs as binary predictors of YOAD is ranked as miR-451a > miR-16-5p > miR-125b-5p > miR-605-5p according to AUC.

For LOAD, the same ROC analysis was performed as stated above and summarized in Fig. 4b. Results for miR-125b-5p showed modest improvement at distinguishing LOAD from HC (AUC = 0.785; CI = 0.537−0.950) than with YOAD. The miR-451a showed high discriminatory potential to distinguish LOAD from HC (AUC = 0.847; CI = 0.679−0.956), but was not as robust as in YOAD. In the case of miR-605-5p, performance at distinguishing LOAD from HC (AUC = 0.765; CI = 0.491−0.913) was similar as in YOAD. Therefore, employing these miRNAs as binary predictors for LOAD suggests a performance ranking of miR-451a > miR-125b-5p > miR-605-5p.

### Linear Combinations of Validated miRNAs Show Synergistic Performance Distinguishing YOAD or LOAD from Healthy Controls

Next, combinations of the validated miRNAs were compared using logistic regression with binary classification and k-fold cross validation to assess the synergistic performance for distinguishing YOAD and LOAD from HC. The following logistic regression results are summarized in Table 2. The combination of all four predictors of YOAD relative to HC (miR-16-5p, miR-125b-5p miR-451a, and miR-605-5p) resulted in an AUC = 0.976, CI = 0.860−0.995, and cross-validated AUC = 0.962. Combining the two best-performing miRNAs at distinguishing HC from YOAD, including miR-451a and the YOAD-specific exosomal miR-16-5p, resulted in a cross-validated performance of AUC = 0.946, CI = 0.807−0.987, and CV-AUC = 0.926. For LOAD versus HC, the combination of the three validated miRNAs (miR-125b-5p, miR-451a, and miR-605-5p) resulted in an AUC = 0.847, CI = 0.688−0.957, and cross-validated AUC = 0.751. Overall, these results indicate that combining these validated miRNAs improved their individual performance at distinguishing YOAD or LOAD from HC.

**Table 2:** Combinatorial performance of the relative expression of validated miRNAs to distinguish AD from HC

MicroRNA combination	Prediction	AUC	95% CI	CV-AUC
miR-16-5p, miR-125-5p, miR-451a, miR-605-5p	HC versus YOAD	0.976	0.860–0.995	0.9619
miR-16-5p and miR-451a	HC versus YOAD	0.946	0.807–0.987	0.9256
miR-125-5p miR-451a, miR-605-5p	HC versus LOAD	0.847	0.688–0.957	0.751

*Prediction* binary prediction, *AUC* area under the curve, *CI* confidence intervals, *CV-AUC* k-fold cross-validation AUC

### Validated miRNA Share Overlapping Targets and Inferred Regional Distribution

The putative mRNA targets of the four validated miRNAs in YOAD were uncovered using the TargetScan v7.1 algorithm [66]. From this, 1508 mRNA targets for miR-16-5p, 100 targets for miR-125b-5p, 28 mRNA targets for miR-451a, and 4028 mRNA targets for miR-605-5p were found (Supplementary Fig. 5a). Overlap is evident between three or fewer groups of the four miRNAs, but no targets are shared between combinations of all four miRNAs (Supplementary Fig. 5a). For LOAD, no targets overlap for miR-125b-5p, miR-451a, and miR-605-5p together, but pairs of each of these miRNAs do share targets (Supplementary Fig. 5b). To assess brain region and cell-type specific localization of these miRNAs, we employed FunRich v3.0 [68]. From this, mRNA targets were depleted in peripheral blood cells and the choroid plexus, as expected. Importantly, markers of the cerebral cortex, hippocampus, cerebellum, or simply brain were enriched. Although no targets were significantly enriched in cerebrospinal fluid, the percentage of predicted targets overlapping with cerebrospinal fluid was higher for all predicted targets overlapping with the blood, peripheral blood cells, blood vessels, and choroid plexus (Supplementary Fig. 5c). These results suggest predominantly CNS localization of transcripts targeted by the validated miRNAs. Analysis of these miRNAs with miRPath (v3.0) showed that combinations of at least four miRNAs overlapped in pathways such as regulating pluripotency of stem cells, PI3K-Akt signaling pathway, AMPK signaling pathway, adrenergic signaling pathway, neurotrophin signaling pathway, MAPK signaling pathway, and Wnt signaling pathway (data not shown).

### Validated miRNA Targets Converge on Common Pathways with Published Microarray Data from the Posterior Cingulate Cortex of Sporadic YOAD

**Fig. 5:**
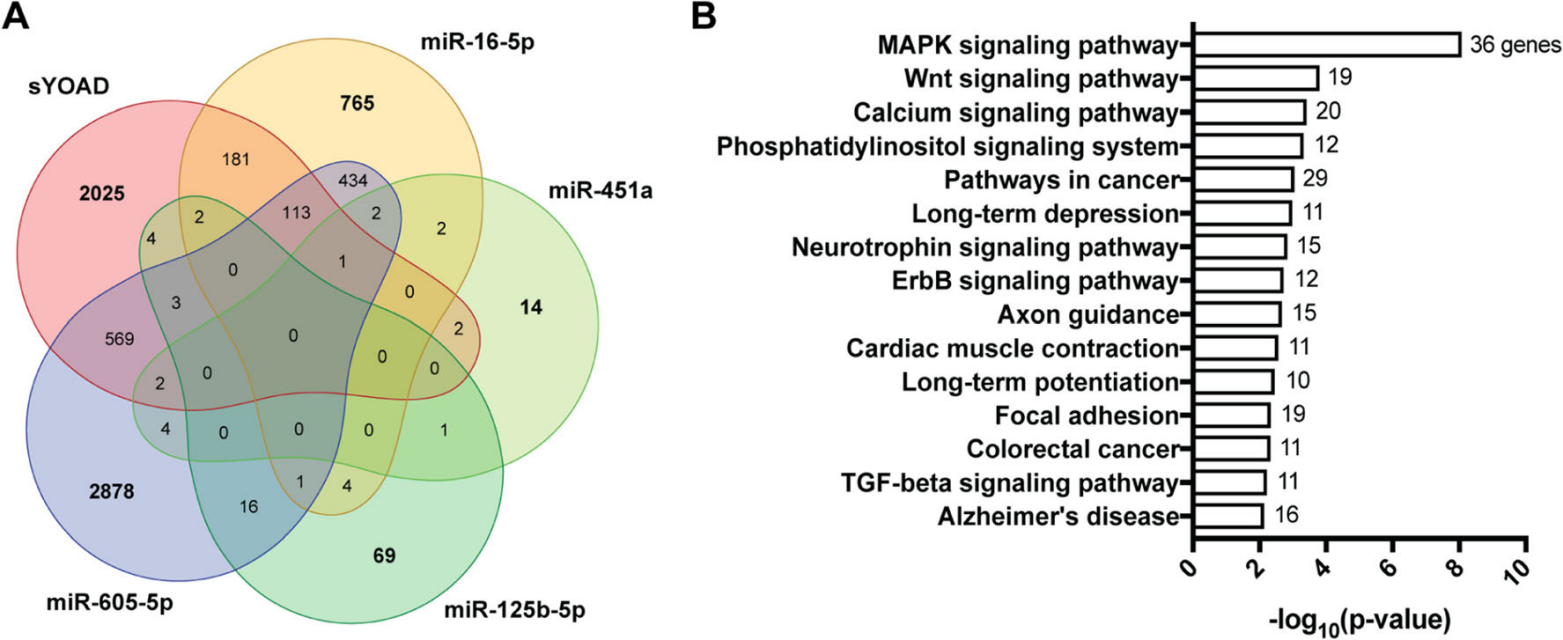
Validated miRNA targets and pathways overlap with sporadic YOAD transcriptome changes. **a** Five-set Venn diagram showing 2899 mRNAs identified as differentially expressed in the posterior cingulate cortex (PCC) overlap of published microarray data (GSE39420). A total of 874 miRNA targets altered in the exosomes of CSF from sporadic YOAD patients overlapped with the entire microarray dataset. **b** KEGG pathways were identified using DAVID (v6.8) with the 874 overlapping validated miRNA targets altered in CSF-derived exosomes from sporadic YOAD. Enriched KEGG pathways (*y*-axis) represented as −log_10_(*p* value) (*x*-axis). The number of genes shared for each pathway is shown at the end of each pathway bar

Published microarray data comparing the transcriptome of the PCC of sporadic YOAD and healthy controls (both *n* = 7) [49] was retrieved from GEO accession ID GSE39420. A differential expression analysis was performed using a published pipeline [70] that was customized in-house in R (v3.4.0). From this, we found that 2899 transcripts were differentially expressed in sporadic YOAD versus control PCC (Fig. 5a; *q* < 0.05). Strikingly, 874 of the 2899 (30.1%) differential transcripts overlapped with the collective targets of our validated miRNAs (Fig. 5a). A summary list of all 399 upregulated and 475 downregulated mRNA targets categorized by validated miRNA is shown in Supplementary Table 4. Using all 874 overlapping transcripts as input into DAVID [73], the top KEGG pathways were found to include MAPK, Wnt, calcium, phosphatidylinositol, neurotrophin, and TGF-beta signaling pathways, as well as long-term depression, axon guidance, long-term potentiation, and Alzheimer’s disease (Fig. 5b; all *p* < 0.05). A substantial overlap of 36 genes in the MAPK signaling pathway was observed (Fig. 5b; FDR < 0.01).

**Fig. 6:**
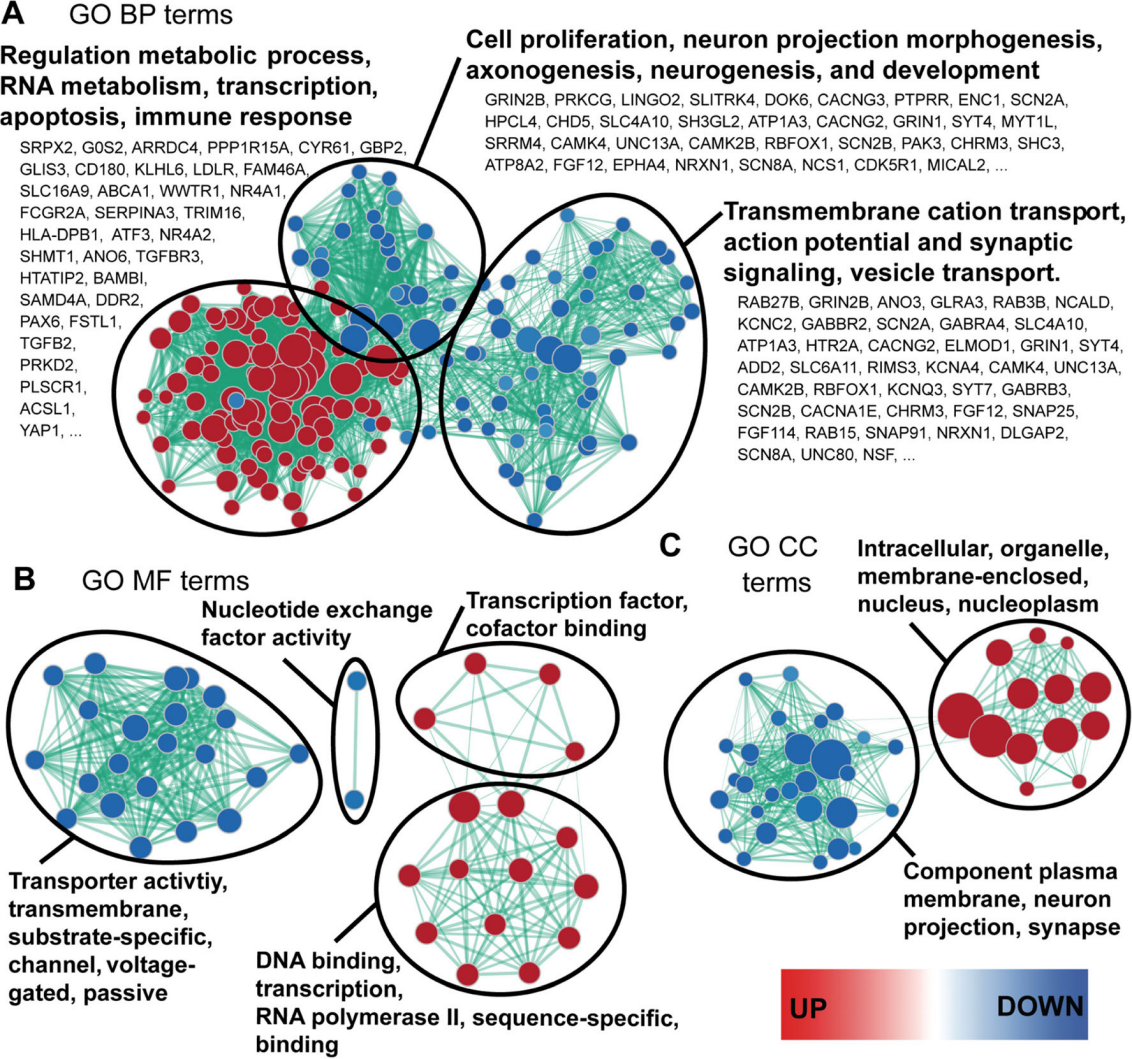
Validated miRNA targets share common gene ontologies with transcripts differentially expressed in the posterior cingulate cortex of sporadic YOAD versus healthy controls. Enrichment map representing Gene Ontology (GO) terms for **a** biological process (BP), **b** GO molecular function (MF), and **c** GO cellular compartment enriched in the 883 overlapping targets differentially expressed in the PCC of sporadic YOAD. For the enrichment maps, red nodes = enriched in sporadic YOAD class, blue nodes = depleted in the sporadic YOAD class. Node colors are scaled based on enrichment significance. Encircled node clusters were selected using the Cytoscape (v3.4.0) plug-in AutoAnnotate (v1.1.0). Overrepresented GO terms shown in bold were selected using the WordCloud (v3.1.0) plug-in based on the proportion of redundancy between node clusters. Lists of transcripts shown under enriched GO BP terms are ranked and ≥ 2 fold change (top upregulated transcripts shown next to red nodes and downregulated transcripts next to blue nodes). False discovery rate (FDR) = *q* < 0.001. Gene sets were pre-ranked using Gene Set Enrichment Analysis (GSEA)

In order to probe for gene ontologies relevant to the 874 overlapping targets altered in YOAD PCC, a GSEA was performed [75]. Visualization of the GSEA results was performed with the Cytoscape plug-in Enrichment Map [76], uncovering a network of both enriched and depleted GO BP, MF, and CC terms (Fig. 6). Top enriched GO BP terms included the regulation of metabolic process, RNA metabolism, transcription, apoptosis, and immune response (Fig. 6a, red node cluster). In contrast, depleted GO BP terms segregated into two distinct clusters. The first cluster related to transmembrane cation transport, action potential and synaptic signaling, and vesicle transport (Fig. 6a, right cluster of blue nodes). The second cluster related to GO BP terms such as cell proliferation, neuron projection morphogenesis, and axonogenesis (Fig. 6a, top cluster of blue nodes). A fold-change-ranked list of transcripts increased in the PCC is provided for GO BP terms, whereby top upregulated terms are shown next to red nodes and downregulated transcripts are shown beside blue nodes (Fig. 6a). If mRNA target repression by altered miRNAs is assumed, upregulated transcripts would be the result of decreased miRNA expression (miR-16-5p, miR-451a, miR-605-5p) and downregulated transcripts the result of increased miR-125b-5p. For GO MF, enriched terms were related to DNA binding, transcription, RNA polymerase II, sequence-specific, transcription factor, and cofactor binding (Fig. 6b, red node clusters). Depleted GO MF terms included nucleotide exchange factor activity, transporter activity, transmembrane, substrate-specific, channel, voltage gated, and passive binding (Fig. 6b, blue node clusters). In agreement with the cellular localization of the GO BP and MF terms reported above, the enriched GO CC terms included intracellular, organelle, membrane-enclosed, nucleus, and the nucleoplasm (Fig. 6c, red node cluster). In addition, the depleted GO CC terms included component of plasma membrane, neuron projection, and synapse (Fig. 6c, blue node cluster).

## Discussion

The current study revealed four miRNAs with altered expression in exosomes derived from CSF of YOAD patients: miR-16-5p, miR-125b-5p miR-451a, and miR-605-5p. In a cohort of LOAD patients, differential expression of miR-125b-5p, miR-451a, and miR-605-5p was also observed. The fact that miR-16-5p was unchanged in LOAD suggests that altered exosomal expression of miR-16-5p may differentiate YOAD from LOAD. Moreover, all four miRNAs altered in YOAD share putative targets and pathways relevant to the post-mortem YOAD brain transcriptome. Intriguingly, these mRNAs were overrepresented in previously implicated pathways in AD, including long-term potentiation [83, 84, 85], MAPK signaling [86–88], Wnt signaling [89, 90], axon guidance [91, 92], and calcium signaling pathway [93–97]. Further, the upregulation of GO terms related to apoptosis and immune response also recapitulates known alterations in AD [98–101]. An overrepresentation of AD-relevant pathways suggests that these miRNAs are potentially combinatorial candidates for understanding disease mechanisms underlying AD. Considering that three of the four validated miRNAs are downregulated, this implicates the absence of target repression [102] by these miRNAs in the enriched pathways. In contrast, the opposite miRNA/mRNA regulatory relationship may be true for upregulated miR-125b-5p.

The significant increase in miR-125b-5p we observed in both YOAD and LOAD patients is in agreement with previous findings in LOAD showing an increase in several CNS structures [103–106] and in CSF [40, 43]. Other reports have shown a decrease of miR-125b-5p in LOAD serum [47, 107] and in CSF [47, 108]. To our knowledge, we are the first to report increased expression levels of miR-125b-5p in CSF-derived exosomes from YOAD and LOAD patients. There is some evidence that miR-125b-5p has a microglial localization and may play a pro-inflammatory role [109]. Moreover, miR-125b-5p overexpression results in hyperphosphorylation of tau and neurotoxicity in primary neuron cultures [103]. However, future studies are required to further examine these mechanisms in the context of AD.

A striking depletion of miR-451a in both YOAD and LOAD was noted in the current study. Previous reports have shown miR-451a to be decreased in the hippocampus [40], temporal cortex [110], and CSF [40] in AD. MiR-451a has a potential role in AD pathogenesis through attenuation of *ADAM10* expression [111]. It appears that miR-451a is unchanged in LOAD serum [112] and is enriched in neuron expression profiles [113], suggesting that miR-451a loss in AD is a CNS-specific molecular signature. Since miR-451a is also decreased in amyotrophic lateral sclerosis [114] and major depressive disorder [115] and plays a role in peripheral inflammation [116], future experiments will confirm whether miR-451a is a general marker of neurodegeneration and/or neuroinflammation.

A previous microarray study reported a modest increase of miR-605-5p in blood mononuclear cells in LOAD [117], but the authors state that miR-605-5p has low expression in the blood. In the whole CSF fraction, Riancho and colleagues (2017) demonstrated expression in 9/10 HC and 9/10 LOAD samples [41], suggesting that miR-605-5p could be specifically altered in CSF-derived exosomes. The organ-specific function of miR-605-5p is not well established in the literature. One group has carefully shown that miR-605-5p promotes the P53 stress response and inhibits apoptosis in colorectal carcinoma cells [118]. Another report recently showed evidence that miR-605-5p acts as a tumor suppressor in melanoma by inhibiting INPP4B [119]. Future studies will explore whether there a CNS-specific role for miR-605-5p exists.

A drawback to our experimental pipeline is the exclusion of a discovery panel on the whole CSF fraction to act as a direct comparison to the CSF-derived exosomes. Hence, future studies will be required to show whether the miRNAs we identified are specifically altered in exosomes alone or are also in other compartments. One previous study suggested that both the extracellular Argonaute2-bound miRNA profile and the exosomal fraction are important, but they found the AUC was higher when the exosomal microRNA was used versus the Argonaute2 microRNA and so concluded that exosomal microRNA may in some way more accurately reflect the pathophysiology of temporal lobe epilepsy and status epilepticus [120]. Despite this limitation, our results partially compare to a recent study exploring the miRNA expression profile in the CSF-derived exosomes from LOAD patients [41]. For example, the study of Riancho and colleagues showed that miR-598 was not detected in the whole CSF fraction of AD patients but was detectable in the majority of CSF-derived exosome preparations. We detected miR-598 in all HC and YOAD patients in the current study, with no difference in relative expression between HC and YOAD. Riancho and colleagues also found that miR-9-5p was more likely to be detected in LOAD; however, we only detected this miRNA in 1/12 HC and 4/17 YOAD samples. Although, we used the same Exiqon exosome extraction kits and these differences could be attributable to CSF input volume (300 μl in Riancho et al. versus 1 ml in our study). Indeed, recent stoichiometric comparison of three commercial precipitation exosome extraction techniques versus ultracentrifugation [121] and subsequent miRNA cleanup [30] demonstrated the relationship between CSF volume input and the exosome particle quantity in the yield. The polymer-based exosome isolation kits demonstrate high yield and overlapping particle size distribution regardless of input [121]. However, the co-precipitation of contaminants in the form of membrane fragments, aggregated proteins, lipoprotein complexes, and/or ribonucleoprotein particles represents caveats to the use of synthetic polymer buffers [122, 123].

We observed a decrease in miR-16-5p in the exosomes from CSF in YOAD. It is known that miR-16 is also a member of the miR-15/107 gene family, targets AD-specific mRNAs, and has been explored as a potential therapeutic target in early AD [124]. Functional studies have uncovered miR-16-5p-mediated inhibition of *APP*, *BACE1*, *MAPT*, and *NICASTRIN* transcripts [124, 125] indicating that AD-relevant transcripts are directly regulated by miR-16-5p. Our findings here agree with previous observations showing a decrease of miR-16-5p in younger LOAD patient CSF compared to controls [108] and in older LOAD patient serum [44] and miR-16-2 in the CSF-derived exosomes from younger LOAD patients [46]. Previous findings also showed that miR-16-5p is decreased in LOAD hippocampus but not in CSF [42] and its decrease correlated with early Braak staging [44].

Interestingly, we did not observe a decrease in miR-16-5p in the exosomes of CSF from our LOAD cohort. This finding may be attributable to differences in the profile of CSF-derived exosomes versus whole CSF, serum, and/or brain tissue. Another possibility is that there may be age-dependent factors contributing to miRNA expression in CSF-derived exosomes. The average age of the LOAD cohort at the time of LP in the current study (75.5 ± 4.6 years) versus the previous CSF study (69.5 ± 7.3 years) differs [108]. In addition, differences exist between the average age of our LOAD and HC cohort (66.5 ± 7.7 years). Future studies will consider the longitudinal change of miR-16-5p expression and other miRNAs in AD CSF, serum, and brain tissue. Also, replication of these results in independent and other neurodegenerative disease cohorts will confirm whether a decrease in CSF exosome levels of these miRNAs is specific for younger AD patients.

Overall, this study uncovered a differential expression profile for both previously identified and novel miRNAs altered in AD and extended these findings to the exosomal compartment in CSF from YOAD and LOAD patients. The expression level of all four miRNAs effectively discriminates YOAD from HC, suggesting the potential for combinatorial value for detection of YOAD versus HC. Since these miRNAs target transcripts and pathways relevant to molecular processes underlying AD and provide some further understanding into the pathophysiological differences between YOAD and LOAD, further functional characterization of these miRNAs may offer new therapeutic avenues for patients with AD.

## Electronic Supplementary Material

ESM 1 (PDF 4130 kb)

Supplementary Table 1 (XLSX 8.77 kb)

Supplementary Table 2 (XLSX 9 kb)

Supplementary Table 3 (XLSX 23 kb)

Supplementary Table 4 (XLSX 20 kb)
