# Supplementary material for: MicroRNA Expression Levels Are Altered in the Cerebrospinal Fluid of Patients with Young-Onset Alzheimer’s Disease
Source: Mol Neurobiol. 2018 Mar 30;55(12):8826–41. doi: 10.1007/s12035-018-1032-x (PMC6208843; doi:10.1007/s12035-018-1032-x)

Supplementary Material

Supplementary Figure Captions

Figure S1. Raw expression data for miRNAs selected for discovery phase normalization. The spike-ins (A) UniSp6 and (B) cel-miR-39-3p and the endogenous (C) hsa-miR-204p-5p were combined using the geometric mean (D) to normalize all miRNAs in the discovery phase. The geometric mean was calculated using the cubed route of the multiplied product of the spike-ins and endogenous miRNA, as previously described [48, 49].

Figure S2. Summary of the qrt-PCR efficiency and melt peaks for validated miRNAs. The qrt-PCR efficiency plots (left) and melt peak curves (right) for (A) hsa-miR-204-5p; (B) hsa-miR-16-5p; (C) hsa-miR-125b-5p; (D) hsa-miR-451a; (E) hsa-miR-605-5p. For the efficiency plots, the x-axis is calculated by taking the negative base 10 logarithm for each of the five serial dilutions of cDNA template; y-axis is the resulting raw Ct value averaged over triplicates (healthy controls). For the melt peaks, the y-axis represents the derivative of the fluorescent reporter intensity versus melting time as a function of temperature (°C) (y-axis). Melt peak overlays are drawn from a subset of qrt-PCR validation reactions.

Figure S3. Raw cycle threshold data for validation phase normalization. The spike-ins (A) UniSp6 and (B) hsa-miR-204p-5p were combined by calculating the geometric mean (C) to normalize miRNA expression. The geometric mean was calculated using the cubed route of the multiplied product of the spike-ins and endogenous miRNA, as previously described [48, 49].

Figure S4. Pearson correlation analysis of relative miRNA expression versus age of lumbar puncture. (A) Correlation plots of miR-16-5p, miR-125b-5p, miR-451a, and miR-605-5p for healthy controls (HC). (B) Correlation plots of miR-16-5p, miR-125b-5p, miR-451a, and miR-605-5p for YOAD. (C) Correlation plots of miR-16-5p, miR-125b-5p, miR-451a, and miR-605-5p for LOAD. R^2 = the square of the Pearson correlation coefficient. Significance was set at $p<0.05$.

Figure S5. Targets predicted for validated miRNAs reveals shared putative mRNA targets. Targetscan v7.1 was used to predict human gene targets for each miRNA altered in either YOAD or LOAD. (A) 4-set Venn diagram overlap of mRNA targets altered in YOAD (hsa-miR-16-5p, hsa-miR-125b-5p, hsa-miR-451a, and hsa-miR-605-5p). (B) 3-set Venn diagram of mRNA targets for miRNAs altered in LOAD (hsa-miR-16-5p, hsa-miR-125b-5p, and hsa-miR-605-5p). (C) Tissue site of expression determined using FunRich v3.0 (http://funrich.org/), which combines multiple established databases incuding UniProt, Human Protein Atlas, Human Proteome Browser, Human Proteome Map, ProteomicsDB, and Human Proteinpedia. Both the percentage of targets showing regional enrichment (blue bar) and the significance of regional enrichment (green bar). Top x-axis is –log10(p-value) of regional enrichment; bottom x-axis is the percentage of targets overlapping with the region.

Supplementary Figures

Supplementary Figure 1

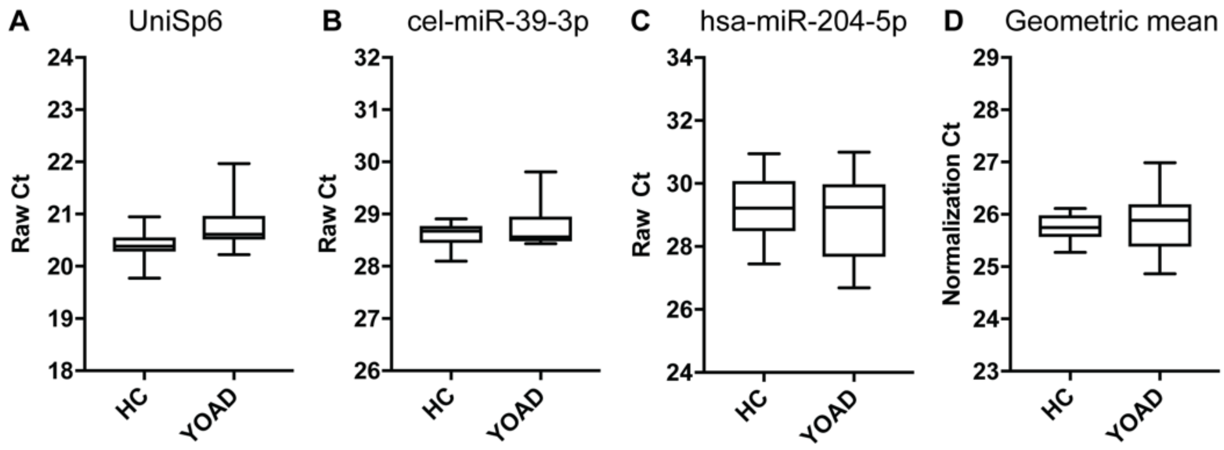

Supplementary Figure 2

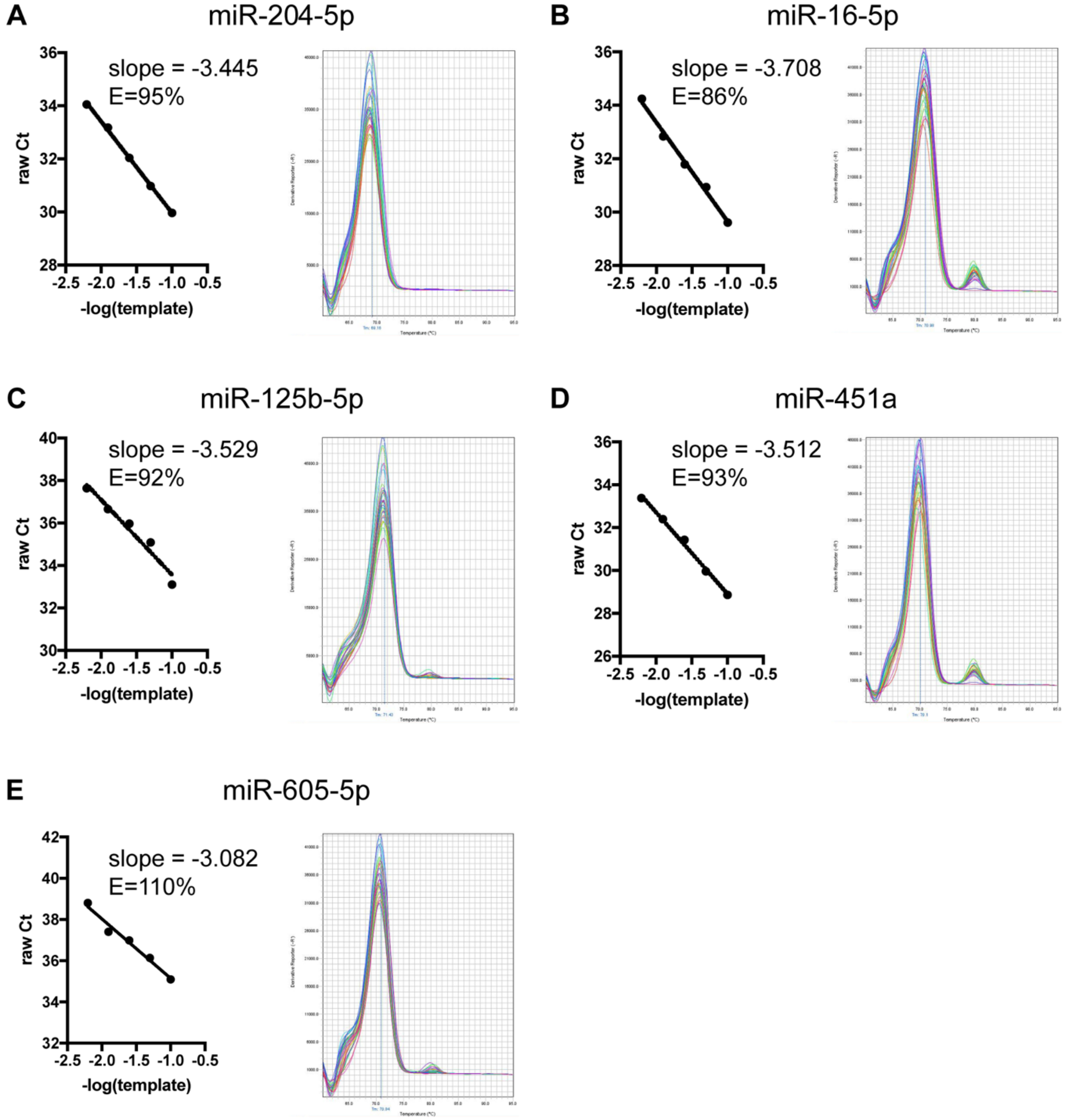

Supplementary Figure 3

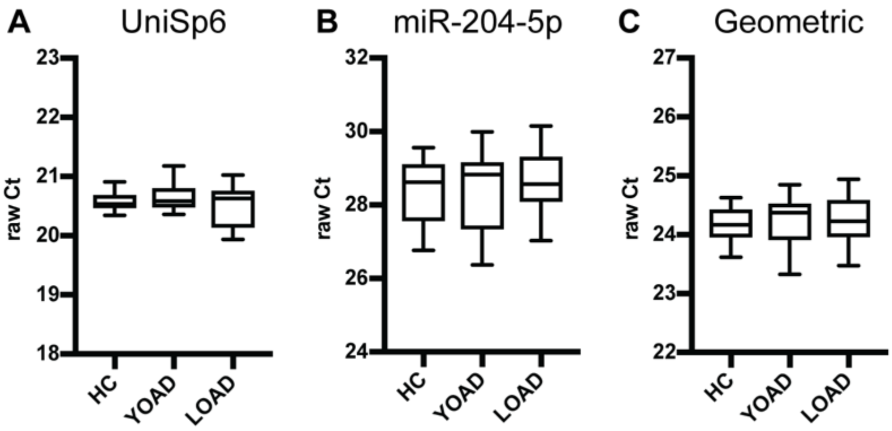

Supplementary Figure 4

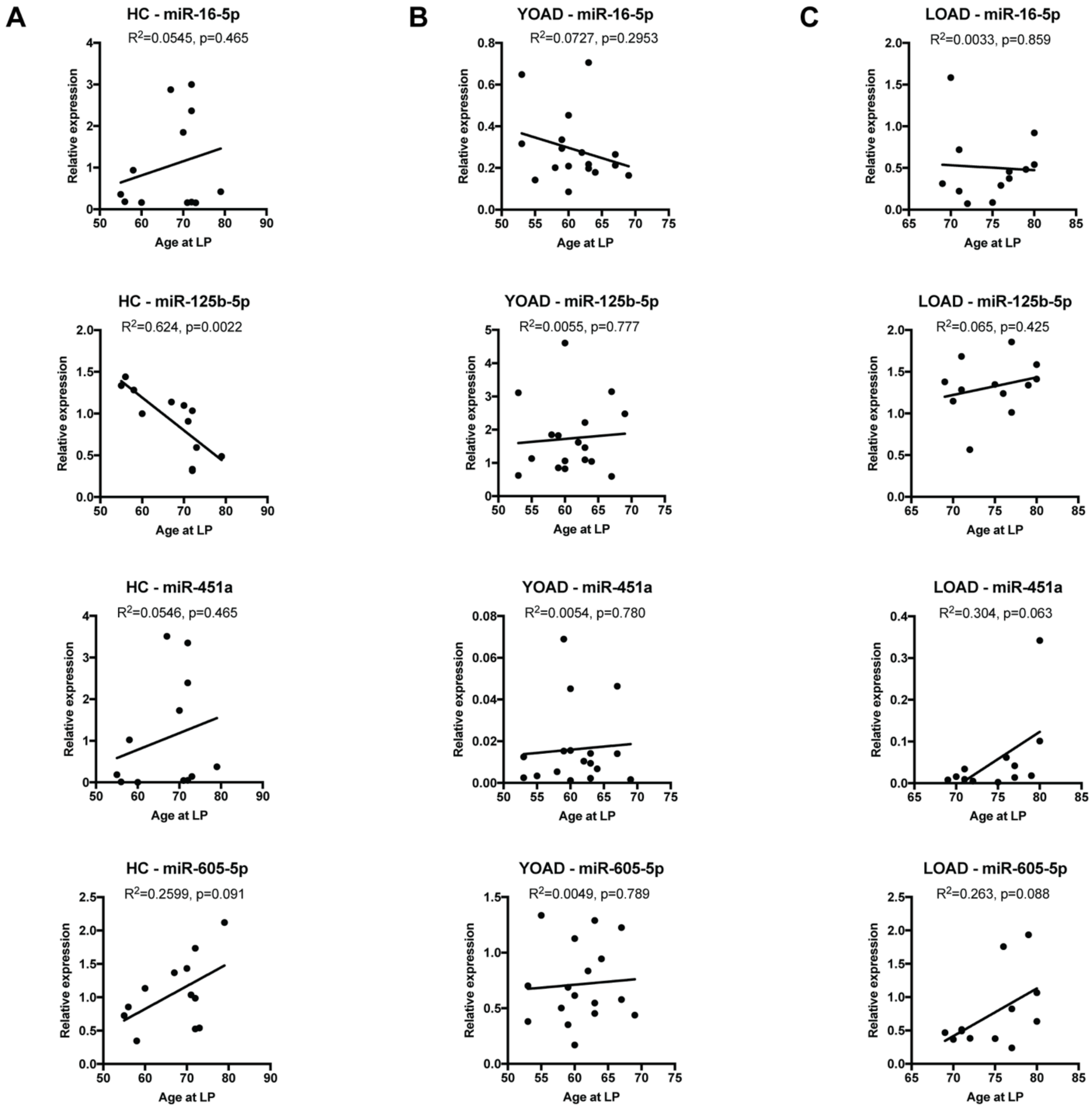

Supplementary Figure 5

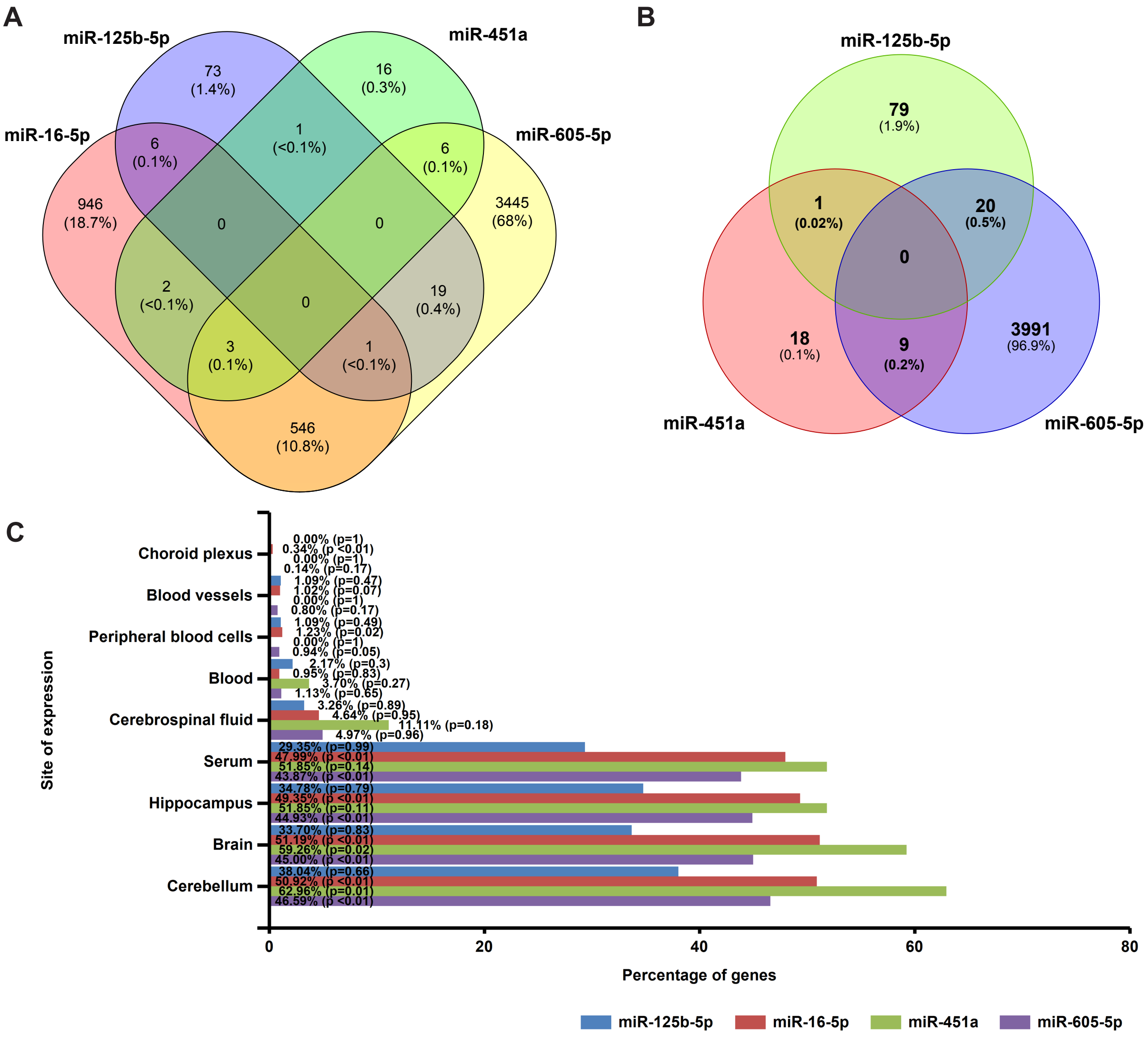

Supplement: Supplementary file 1 — (PDF 4130 kb) [file 12035_2018_1032_MOESM1_ESM.pdf]
